# Plasma hepatocyte growth factor as a noninvasive biomarker in small cell lung cancer

**DOI:** 10.1186/s12885-023-10995-z

**Published:** 2023-10-12

**Authors:** Cong Zhao, Li Tong, Bin Liu, Fei Qi, Zhiyun Zhang, Yi Guo, Yanxia Liu, Ying Wang, Lina Zhang, Baohua Lu, Baolan Li, Tongmei Zhang

**Affiliations:** 1 General Department, Beijing Chest Hospital, Capital Medical University, Beijing, 101149 China; 2 Department of Oncology, Beijing Chest Hospital, Capital Medical University, Beijing, China; 3 Cancer research center, Tuberculosis and Thoracic Tumor Research Institute, Beijing Chest Hospital, Beijing, China; 4 Emergency Department, Dongfang Hospital Beijing University of Chinese Medicine, Beijing, China

**Keywords:** Hepatocyte growth factor, Plasma, Pan-cancer analysis, Small cell lung cancer, Metastases

## Abstract

**Background:**

Hepatocyte growth factor (HGF) is a peptide-containing multifunctional cytokine, which is overexpressed and/or activated in multiple malignancies and is reported to be associated with tumor development and inferior survival. At present, the role of HGF in small cell lung cancer (SCLC) has not been fully explored yet.

**Materials and methods:**

The expression of HGF and its value in predicting survival in SCLC were explored from GEO database and in pan-cancer analysis. Furthermore, we detected the expression of HGF using tumor tissue and paired plasma samples from a validation cohort of 71 SCLC patients at our institute. Correlation between tumor and plasma HGF expression and the prognostic values were analyzed.

**Results:**

GEO database analysis revealed that tumor tissue had lower HGF expression than paired normal tissue in SCLC. At our institute, immunohistochemical staining showed negative expression of HGF in tumor tissue of SCLC at our institute (47/47, 100%). The average baseline plasma HGF was 1.28 (range,0.42–4.35) ng/ml. However, plasma HGF was higher in SCLC patients with patients with N_3,_ M_1_, liver metastasis (LM) and bone metastasis (BM) disease compared with those N_0 − 2_ (1.25 vs. 1.75 ng/mL, *P* = 0.000), M_0_ (1.26 vs. 1.63 ng/mL, *P* = 0.003), non-LM (1.32 vs. 2.06 ng/mL, *P* = 0.009), and non-BM (1.35 vs. 1.77 ng/mL, *P* = 0.047), respectively. Multivariate analysis revealed plasma HGF was an independent predictor for LM and prognostic factor of OS.

**Conclusion:**

Our results revealed that plasma HGF rather than tumor HGF exhibited a potential role in predicting metastasis and survival in SCLC. Plasma HGF might be used as a non-invasive detecting and monitoring tool for SCLC.

**Supplementary Information:**

The online version contains supplementary material is available at 10.1186/s12885-023-10995-z.

## Introduction

SCLC is identified as one of the most deadly malignancies with limited therapeutic efficacy worldwide [1]. Patients especially those with extensive stage disease progress rapidly with a median OS of only 6–8 months [2–4]. For the last few decades, as drug after drug has failed and fallen due to little impact on progression-free survival (PFS) or OS, SCLC has been notorious for its lack of progress [5, 6]. Therefore, it is in urgent need to discover novel biomarkers with therapeutic potential for SCLC at present.

In several tumors, MET/HGF axis is aberrantly activated and represents one of the most important mechanisms of progression and invasiveness, which is proved to promote proliferation, migration, invasion, angiogenesis and tumorigenesis. It is also demonstrated to be associated with drug resistance and inferior survival for cancer patients [7–11].

HGF, secreted from stromal and mesenchymal cells, is a peptide-containing multifunctional cytokine that acts on various epithelial cells to regulate cell growth, morphogenesis, and organizing multistep of angiogenesis in many organs [12]. Apart from HGF detected in tumor tissue, elevated HGF in blood expression was associated with high risk for metastasis and inferior OS in breast, bladder, gastric, esophageal, colorectal cancers, ovarian cancer and myeloma [13–17].

Previous studies find that the MET/HGF axis appears to be a signaling pathway frequently altered in SCLC [18–20]. Rygaard et al. concluded that the MET/HGF axis was frequently active in SCLC, possibly by a paracrine regulatory pathway of HGF [21].

Paracrine of HGF maybe influence the level of plasma HGF, which maybe associated with prognosis of SCLC. The role of plasma HGF in SCLC, however, has not been systematically explored so far. Since few data on SCLC is avaliable from on-line database, we explore the expression of HGF in pan-cancer and analyzed its potential predictive value. Furthermore, using an independent cohort of 71 SCLC patients at our institute, we identified HGF expression in tumor tissue and paired plasma and also explored the potential use of plasma HGF in predicting metastasis and prognosis. We found that plasma HGF might be used as a high sensitivity and specificity monitoring tool for metastasis and survival in SCLC, establishing theoretical basis for clinical decision-making.

## Materials and methods

### Pan-cancer analysis of HGF expression and its prognostic value

The gene expression of HGF were downloaded and recomputed via the Sangerbox 3.0 web (http://vip.sangerbox.com/home.html) from the cancer genome atlas (TCGA) and genotype-tissue expression (GTEx) [22]. All expression data were normalized via log2 conversion. R (version 4.2.0) was used to investigate the expression of HGF between different cancers and corresponding adjacent tissues or normal tissue. The genetic variations of HGF were analyzed using the cBioPortal tool (https://www.cbioportal.org/, accessed on 20 June 2021) [23]. We selected the “TCGA Pan Cancer Atlas Studies” module in cBioPortal, and then entered the HGF gene to query the cancer types summary, and obtained the alteration frequency. Survival analysis and K-M plotter were used to analyze the prognosis value of HGF, regarding to OS and progression-free interval (PFI).

### Detection of DEGs in SCLC based on GEO database

The differentially expressed genes (DEGs) between SCLC and adjacent normal lung tissues were screened out using EdgeR. Samples were divided into HGF^low^ and HGF^high^ groups using a cutoff value of median expression level. DEGs between them were screened out using EdgeR of R. DEGs were defined with FDR < 0.05 and |logFC| > 1.

### Validation of HGF expression and its potential role in SCLC at our institute

#### Study design and participants

This was an exploratory retrospective study of 71 consecutive patients who were diagnosed with SCLC between December 2017 and April 2020 at our institute. Inclusion criteria: pathologically diagnosed with SCLC; available plasma samples at diagnosis for enzyme linked ELISA detection. Patients with insufficient samples, incomplete record, or lost follow-up were excluded. Clinicopathological data including age, sex, smoking history, TNM stage, metastasis, serum carcinoembryonic, treatment and others were collected. This study was approved by the Ethics Committee of Beijing Chest Hospital.

#### Sample collection

A total of 71 frozen plasma samples and 47 tumor specimens were included and stored in the refrigerator at -80℃. Tumor specimens were collected using core needle biopsies, endobronchial biopsies, or tissue resection.

#### Detection of HGF in plasma and tumor tissue

71 samples were selected for HGF ELISA. 6ml peripheral venous blood was drawn into Ethylene Diamine Tetraacetie Acid (EDTA)anticoagulant tube. Samples were centrifuged for 2 h (3500 rpm/min, 5 min) to separate plasma, which were stored in the refrigerator at − 80℃. Enzyme linked immunosorbest assay (ELISA) was used to measure plasma HGF (ELISA kits, USCN Life Sciences, Wuhan, China, Code: BAH-MET-HGF-1) and an ELISA reader (Immunoscan, BioTek Instruments, Ins, USA). The evaluation was performed according to the manufacturer’s protocol and published studies [19, 20]. IHC staining was carried out on formalin-fixed, paraffin-embedded (FFPE) tissue sections of tumor specimens. The sections were then reacted with primary antibodies (HGFβ[D6S7D) XP® Rabbit, Cell Signaling, Code: 52,445). The protocol and evaluation were performed according to the manufacturer’s protocol and published studies [24].

#### Statistical analysis

OS was calculated from the date of initial diagnosis to the date of death from any cause, or the last follow-up. PFS was defined as the interval from the date of diagnosis to the date of first progression, death or last follow-up. Data were analyzed using the SPSS 22.0, GraphPad Prism (San Diego, CA, USA) and R software package (version 4.2.0: http://www.Rproject.org). The continuous variables and categorical variables were assessed by the Student’s t test and the Pearson’s chi-square respectively. Multivariate analysis was conducted by logistic regression model and Cox proportional hazards models. A Kaplan–Meier survival analysis in different subgroups was performed. If *P* value in the univariable analysis was less than 0.05, this variable should be adjusted in multivariate analysis. A two-tailed *P* < 0.05 was considered statistically significant in all analyses.

## Results

### HGF expression in SCLC in GEO database

**Fig. 1:**
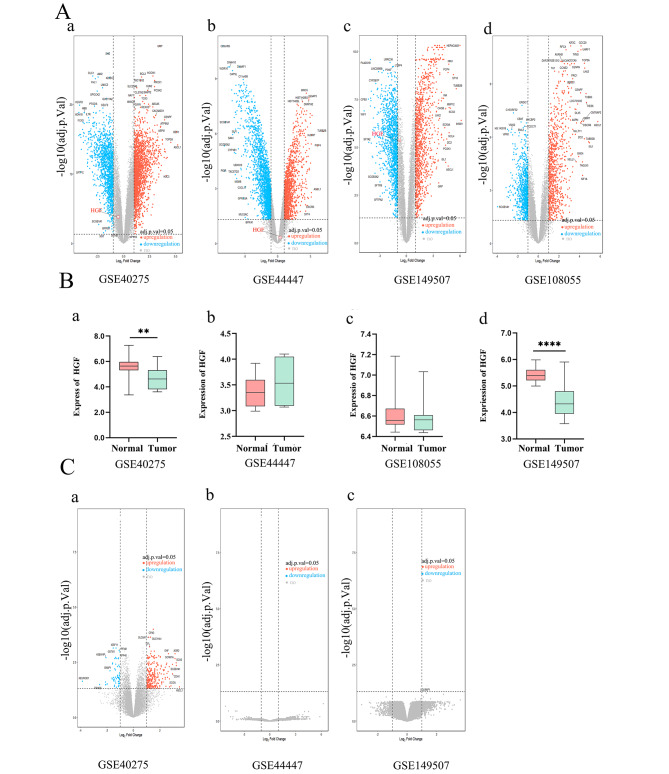
A: The detection of DSE between SCLC and adjacent normal lung tissues in SCLC in GEO, Volcano plot of the expression level of differentially expressed genes in four datasets(a;GSE40275,b:GES4447,c:GSE108055,d:GSE149507). B: The expression level of HGF in SCLC between normal tissue and primary tissue of selected tumors(a;GSE40275,b:GES4447,c:GSE108055,d:GSE149507). C:Volcano plot of the expression level of differentially expressed genes in HGF^low^ and HGF^high^ groups from GSE40275, GSE44447, and GSE149507. Red dots represent a high expression of genes and blue dots represent a low expression of genes (Fig. 1C(a-c)). * *p*<0.05,** *p*<0.01, *** *p*<0.001,**** *p*<0.0001

HGF were significantly downregulated in SCLC specimens in comparison to adjacent normal tissues in GSE40275, GSE108055 and GSE149507 datasets. However, it was upregulated in SCLC in GSE44447, though the difference was not statistically significant (*P* = 0.06, Fig. 1A). DEGs between SCLC and adjacent normal lung tissues were screened out using EdgeR. But according to the threshold values for DEGs identification, HGF was not screened in neither upregulative and downregulative DSEs in GSE40275, GSE44447 and GSE149507 datasets. HGF was not detected in GSE149507 datasets (Fig. 1B). DEGs between HGF^low^ and HGF^high^ groups were shown in Fig. 1C and no significant genes with clinical meaning were sorted out in SCLC. In summary, HGF expression of SCLC was not identical according to different database; besides, its clinical relevance and predictive value in SCLC remained undefined at present.

### Pan-carcinogenic analysis of HGF

**Fig. 2:**
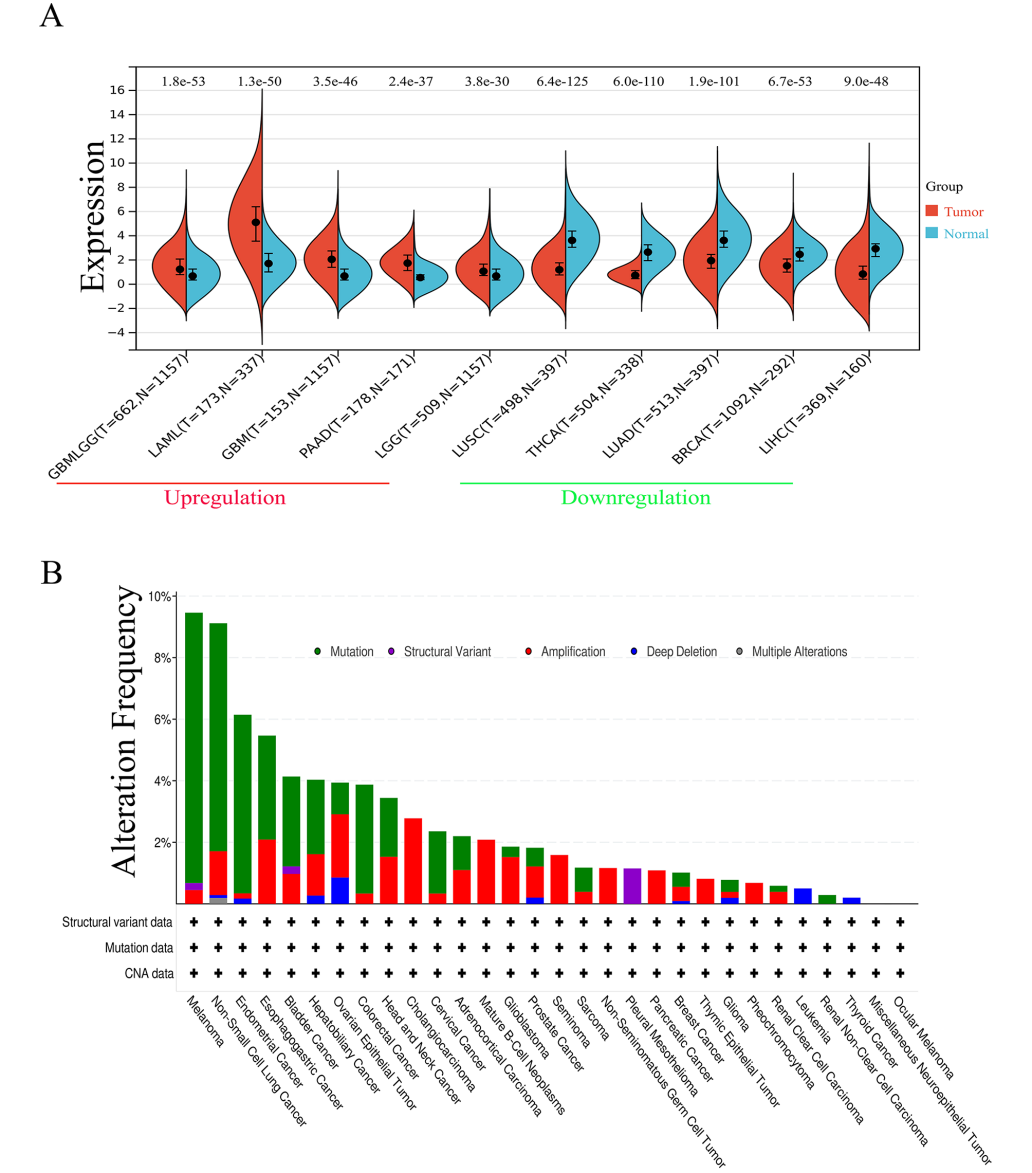
A: Upregulated and downregulated mRNA expression of HGF in pan-cancer. HGF expression was remarkably increased in 5 cancer types above the red line. And the HGF expression was remarkably reduced in 5 cancer types above the green line. B: Genetic alternation of HGF in different tumors, the alteration frequency with mutation type was displayed

**Fig. 3:**
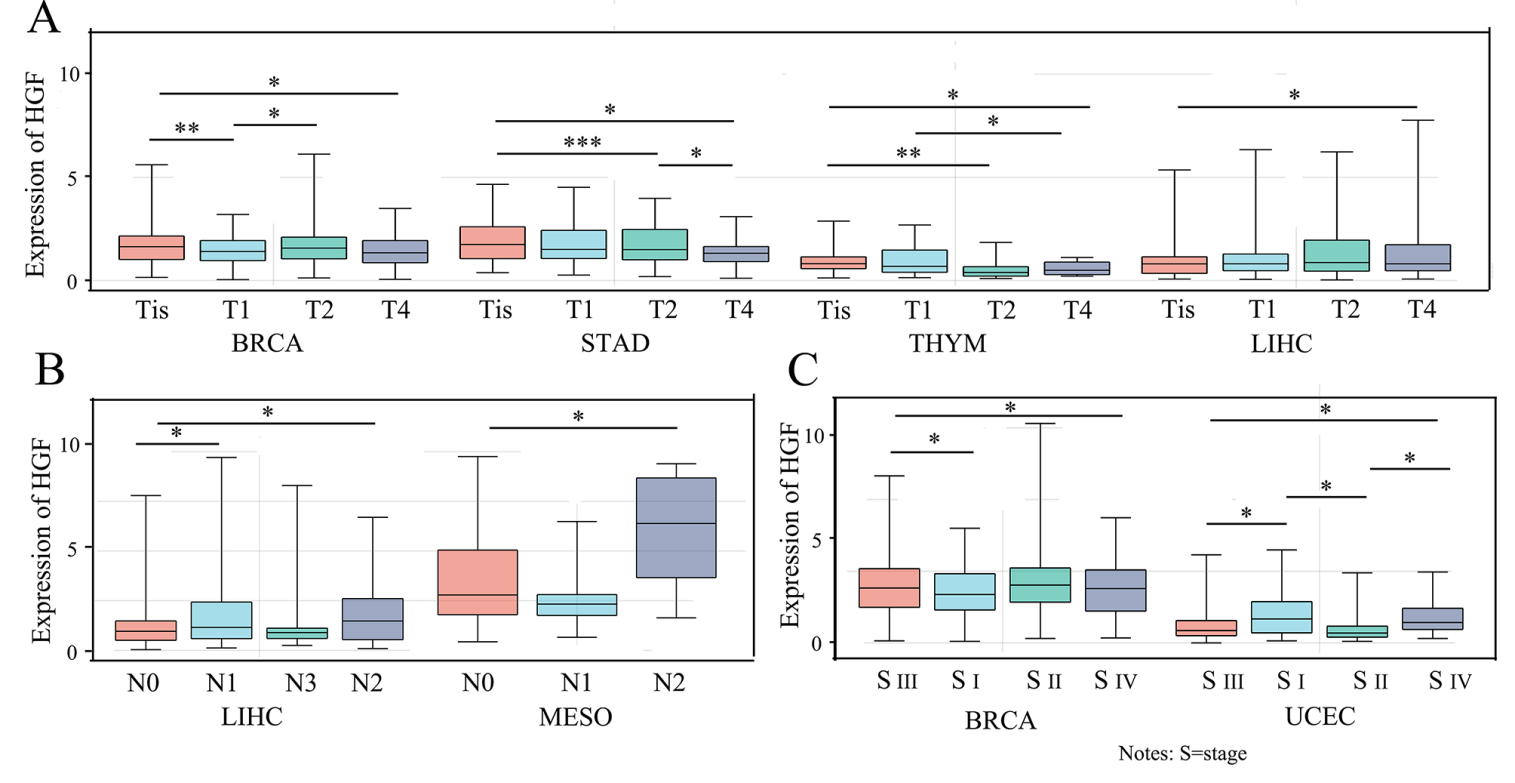
Correlations between the HGF expression and Clinicopathology, were investigated based on the TCGA data. A: T stage of BRCA, STAD, THYM and LIHC, B: N stage of LIHC and MESO, C: the main pathological stages including stage I, stage II, stage III, and stage IV of BRCA and UCEC. Log2 (TPM + 1) was used for log scale. ∗*p* < 0:05, ∗∗*p* < 0:01, and ∗∗∗*p* < 0:001

The regulation of the HGF expression between various types of cancer and normal tissues was investigated from the GTEx database and TCGA. As shown in Fig. 2A, upregulated HGF expression in tumor was observed in some solid tumors. Whereas, it was downregulated in lung squamous cell carcinoma (LUSC), lung adenocarcinoma (LUAD), thyroid carcinoma (THCA), breast invasive carcinoma (BRCA) and liver hepatocellular carcinoma (LIHC). Apart from expression, we observed various genetic alternations of HGF in tumors from TCGA cohort (Fig. 2B). Melanoma and non-small cell lung cancer (NSCLC) had the highest frequency of HGF genetic alteration (> 8%) with mutation as the main variation. HGF expression was significantly associated with tumor burden reflected by stage especially T stage and N stage in some certain types of cancer as shown in Fig. 3A-C. Among BRCA and STAD, T1 disease had higher HGF expression than T4 lesion(Figure 3A). Besides, N1 had higher HGF expression compared to N0 disease in LIHC and MESO (Fig. 3B). HGF expression negatively correlated with stage in BRCA and UCEC (Fig. 3C).

**Fig. 4:**
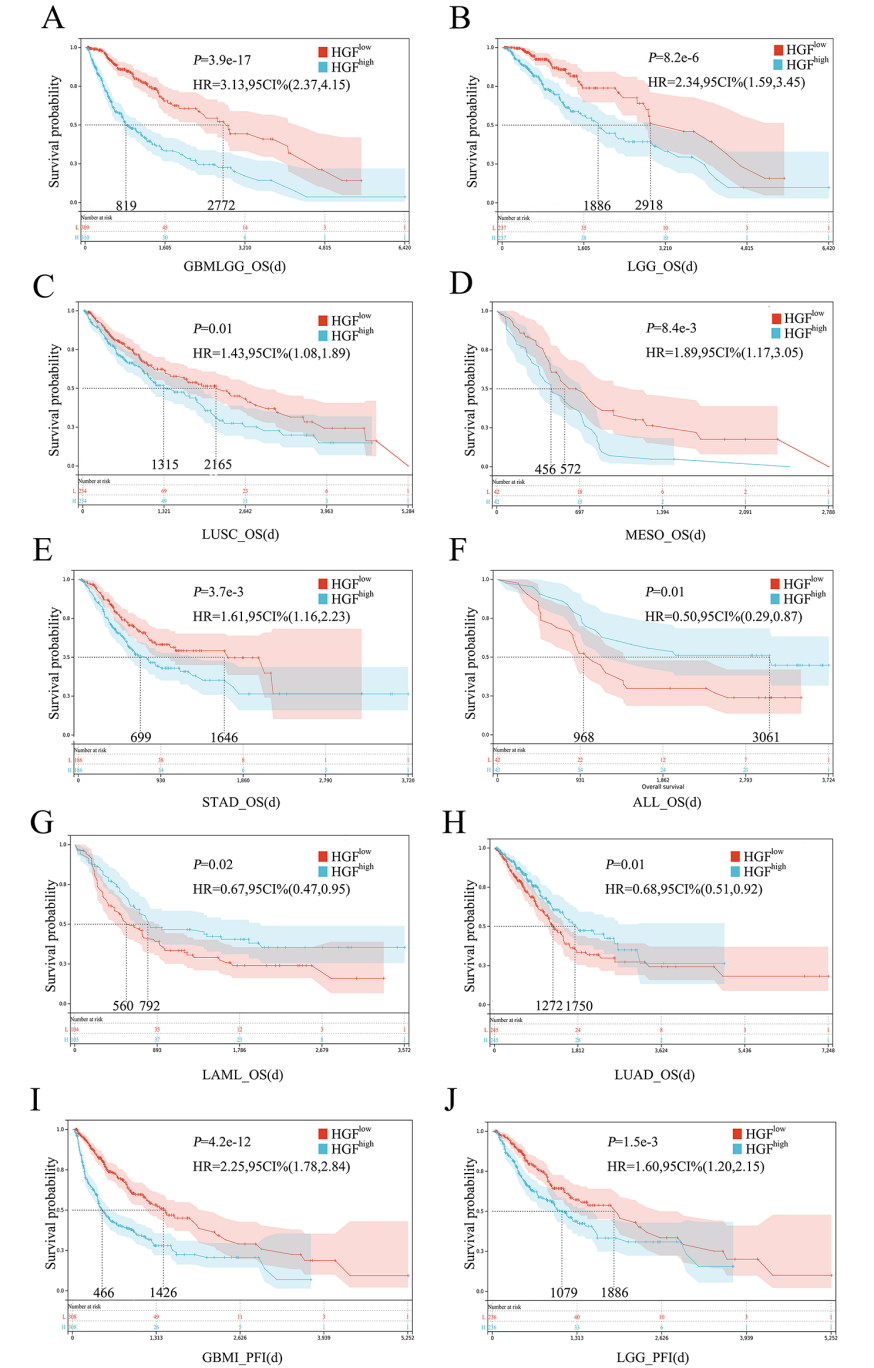
Association between the HGF expression and the prognosis of cancer patients. Kaplan-Meier survival curves of OS for patients stratified by the different expressions of HGF in GBMLGG, LGG, LUSC, MESO, STAD ALL, LAML and LUAD(A-H). And Kaplan-Meier survival curves of PFI for patients stratified by the different expressions of HGF in GBMI and LGG(I-J).

Pan-cancer analysis revealed different prognostic value of HGF expression in various cancer types (Fig. 4A-J). Upregulation of HGF was significantly associated with inferior OS in GBMLGG, LGG, LUSC, MESO and STAD (Fig. 4A-E). In contrast, upregulation of HGF indicated superior OS in ALL, LAML and LUAD (Fig. 4F-H). Likewise, a correlation was also observed that the upregulation of HGF was significantly associated with inferior PFI in GBMI, LGG(Fig. 4I-J).

### HGF expression in tumor and plasma in SCLC at our institute

A total of 71 patients were included in this analysis and the baseline characteristics were summarized in Supplementary Table 1. The patients included 56 male and 15 female patients, Limitation stage 27, extension stage 44. At diagnosis, 13 cases had LM, 18 with BM and 4 with intracranial metastasis (IM). IHC staining on the paraffin-embedded tumor specimens from 47 SCLC patients showed negative expression of HGF in each sample (Supplementary Fig. 1). However, HGF was detected in plasma samples from 71 SCLC patients with a median concentration of 1.46ng/mL.

### Plasma HGF as an indicator for LM in SCLC at our institute

**Fig. 5:**
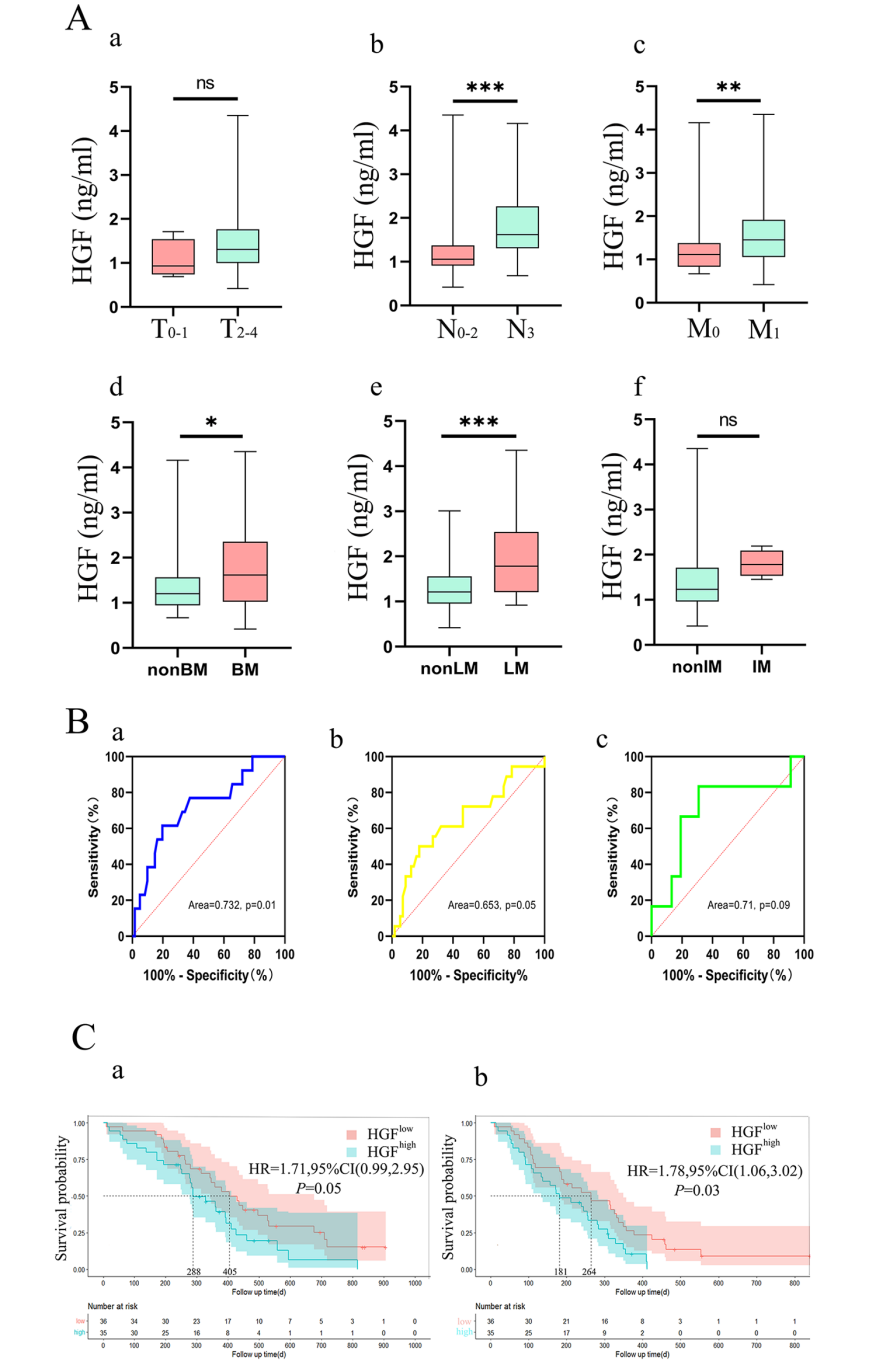
A: Compared the level of plasma HGF in different group (a: T stage, b: N stage, c: M stage, d: LM, e: BM, f:IM). B: Receiver operating curve analysis of metastases (a: LM, b:BM; c:IM). C: Survival analysis of patients with SCLC in the HGF^low^ and HGF^high^ groups (a: PFS, b: OS) ,* *p*<0.05,** *p*<0.01, *** *p*<0.001

Plasma HGF was higher in patients with N_3_, M_1_, LM and BM groups than that in patients with N_0 − 2_ (1.25 vs. 1.75 ng/mL, *P* = 0.000), M_0_ (1.26 vs. 1.63 ng/mL, *P* = 0.003), non-LM (1.32 vs. 2.06 ng/mL, *P* = 0.009), and non-BM (1.35 vs. 1.77 ng/mL, *P* = 0.047), respectively (Fig. 5A b-e). Plasma HGF was higher in T_2 − 4_ and IM groups compared to T_0 − 1_ and non-IM groups, though the difference did not reach statistical significance (both *P* > 0.05, Fig. 5A a,f). ROC curve analysis showed similar results for LM, BM and IM (AUC: 0.723, 0.653 and 0.71, respectively), LM had a largest AUC of 0.73. This suggests that HGF was a potential biomarker for LM (Fig. 5B a-c). Besides, mutivariavte analysis showed HGF as independent predictive factors of LM (OR = 1.499, 95% CI, 1.023–2.196, *P* = 0.038; Table 1).

### Univariate and multivariate analysis of survival in SCLC

Patients were subdivided into HGF^high^ and HGF^low^ group based on a cutoff value of 1.72 ng/mL of plasma HGF, which was calculated via ROC using LM as the point. Till the last visit in April 2022, the median follow-up period reached 10.5 months. A total of 63 patients had recurrence or metastases, 54 cases of which died of cancer-related causes. Patients in HGF^low^ group achieved much more favorable OS (13.5 vs. 9.6 months, *P* = 0.03) and PFS (8.8 vs. 6.0 months, *P* = 0.05) compared with those in HGF^high^ group (Fig. 5C). Univariate analysis showed that plasma HGF were negatively associated with OS (OR = 1.721, 95% CI, 1.213–2.442, *P* = 0.002) and PFS (OR = 1.443, 95% CI, 1.052–1.98, *P* = 0.023). Furthermore, plasma HGF was demonstrated as an independent prognostic factor for OS via multivariate Cox regression (OR = 1.629, 95% CI, 1.110–2.392, *P* = 0.013, Table 2).

**Table 1:** Uni-and multivariate analysis for LM

Parametres	Univariate analysis			Multivariate analysis	
	OR(95% CI)	*p*		OR(95% CI)	*p*
HGF	3.374(1.414–8.054)	0.006		1.499(1.023–2.196)	0.038
Sex	1.589(0.312–8.094)	0.577			
Age	0.783(0.215–2.856)	0.711			
Smoking history	3.467(0.411–29.210)	0.253			
LDH	1.000(0.998–1.001)	0.694			
AST	0.993(0.965–1.022)	0.653			
ALT	0.978(0.940–1.018)	0.282			
ALB	0.902(0.808–1.006)	0.063			
CEA	1.015(0.997–1.034)	0.106			
NSE	1.008(0.998–1.018)	0.101			
SCC	0.594(0.054–6.548)	0.670			
CYFRA21-1	1.251(1.053–1.486)	0.011		1.081(1.016–1.151)	0.014
Pro-GRP	1.001(1.000-1.001)	0.003			

AST: aspartate transaminase; ALT: Alanine aminotransferase; LDH: lactate dehydrogenase; ALB:albumin; CEA: carcinoembryonic antigen ; NSE: neuron-specifific enolase; SCC:Squamous cell carcinoma antigen;CYFRA21-1:Soluble fragment of cytokeratin 19;Pro-GRP: pro-gastrin-releasing peptide

**Table 2:** Uni-and multivariate analysis for OS

Parametres	Univariate analysis			Mutlivariate analysis	
	OR (95% CI)	*p*		OR (95% CI)	*P*
HGF	1.721(1.213–2.442)	0.002		1.629(1.110–2.392)	0.013
Sex	0.588(0.276–1.253)	0.169			
Age	1.374(0.784–2.409)	0.267			
Smoking history	2.171(0.977–4.824)	0.057			
ECOG-PS	3.157(1.475–6.754)	0.003			
T stage	2.765(0.670-11.418)	0.160			
N stage	2.220(1.278–3.858)	0.005			
M stage	2.933(1.669–5.153)	0.000		2.287(1.261–4.150)	0.006
CEA	1.009(1.005–1.014)	0.000		1.008(1.003–1.013)	0.002
NSE	1.005(1.000-1.009)	0.034			
SCC	0.578(0.237–1.411)	0.229			
CYFRA21-1	1.084(1.022–1.150)	0.007			
Pro-GRP	1.000(1.000–1.000)	0.319			

ECOG-PS = Eastern Cooperative Oncology Group performance status; CEA = carcinoembryonic antigen ; NSE = neuron-specifific enolase; SCC: Squamous cell carcinoma antigen;CYFRA21-1 = Soluble fragment of cytokeratin 19;Pro-GRP: pro-gastrin-releasing peptide

## Discussion

In this study, we analyzed the expression and its association with clinical parameters and its influence on survival in pan-cancer based on public database; furthermore, we validated the conclusions using an independent cohort of 71 patients with SCLC at our institute. And to our knowledge, this is a comparably large sampled study which first focuses on HGF expression in both tumor and plasma in SCLC. Our results revealed that plasma HGF rather than tumor HGF might be a reliable biomarker for LM and inferior survival in SCLC, and its potential value for noninvasive disease monitoring needs to be further explored.

SCLC is typically diagnosed in small biopsies or cytology specimens, with routine immunostaining only [1]. Result from GEO database presented that the expression levels of HGF was downregulated in SCLC specimens in comparison to adjacent normal tissues (Fig. 1A). HGF wasn’t DEGs between SCLC and normal tissues (Fig. 1B). What’s more, IHC staining on the paraffin-embedded sections of 47 SCLC at our institute presented negative expression for each patient. Our result was contradictive with some published studies, in which expression of HGF and its receptor c-MET has been reported to be increased in lung, colon, breast, thyroid, renal carcinoma, melanoma and various sarcomas [25, 26]. The heterogeneity of cancers and unique tumor environment might be one reason for this controversy. Therefore, tumor HGF may not be a feasible biomarker for SCLC. Most cancer cells do not express HGF, and HGF is secreted mainly by cells of mesenchymal origin, acts in a paracrine manner on cells that express the c-MET receptor [27]. HGF/c-MET interactions are activated by stromal cell HGF, which produces a suitable microenvironment for cancer cell growth and invasion, and in an autocrine manner by c-MET produced by cancer cells [28, 29]. These studies suggest us that HGF in plasma may play an important biological role in SCLC. In advanced ovarian cancer, HGF in serum was demonstrated as an indicator of poor prognosis [16, 30]. Hitomi Umeguchi and his teamwork [31] showed that HGF in plasma was significantly higher in the advanced stage of cancer and predicted poor survival as determined using 315 plasma samples from 225 lung cancer patients. Consistently, our results showed plasma HGF was demonstrated as an independent predictive factor for LM (OR = 1.499, 95% CI, 1.023–2.196, *P* = 0.038; Table 1) with a ROC of 0.73 for LM diagnosis and an independent prognostic factor for OS (OR = 1.629, 95% CI, 1.110–2.392, *P* = 0.013).

In conclusion, plasma HGF might be of great value as a non-invasive biopsy tool for SCLC. It overcomes the difficulty of obtaining tumor tissue in some certain circumstances. Additionally, it can avoid the risk of bleeding and pain of patients and can be used for dynamic monitoring. As is known to all, little progression on treatment and prognosis is obtained in SCLC in the past three decades, and reliable prognostic predictor is also in lack for SCLC. Therefore, our result that plasma HGF might be a potential predictive factor for LM and OS did provide a promising perspective. However, the true value of plasma HGF in SCLC needs to be further validated in large-sample prospective studies.

### Electronic supplementary material

Below is the link to the electronic supplementary material.

Supplementary Table 1 

Supplementary Figure 1

## Data Availability

The datasets generated and/or analysed during the current study are available in the GEO[https://www.ncbi.nlm.nih.gov/geo] and TCGA [https://www.cancer.gov/about-nci/organization/ccg/research/structural-genomics/tcga]. The clinical data used during the current study available from the corresponding author on reasonable request.

## Declarations

### Supervision,Software, Validation. All authors

Writing,Reviewing and Editing.
