# Supplementary material for: Plasma hepatocyte growth factor as a noninvasive biomarker in small cell lung cancer
Source: BMC Cancer. 2023 Oct 12;23:973. doi: 10.1186/s12885-023-10995-z (PMC10568809; doi:10.1186/s12885-023-10995-z)
Supplement: Supplementary file 1 — Supplementary Table 1 [file 12885_2023_10995_MOESM1_ESM.docx]

Supplementary table 1 Baseline characteristics of the 71 patients enrolled in this study.

| **Parameters** | **N (%)** |
| --- | --- |
| Sex |  |
| male | 56(78.9%) |
| female | 15(21.1%) |
| Age |  |
| ≤65 | 46(64.8%) |
| >65 | 25(35.2%) |
| Smoking history |  |
| no | 14(19.7%) |
| yes | 57(80.3%) |
| ECOG-PS |  |
| ＜2 | 59(83.1%) |
| ≥2 | 10(16.9%) |
| T stage |  |
| ≤1 | 5(7.0%) |
| >1 | 65(91.5%) |
| Unknown | 1(1.5%) |
| N stage |  |
| ≤2 | 42(59.2%) |
| >2 | 29(40.8%) |
| M stage |  |
| no | 34(47.9%) |
| yes | 37(52.1%) |
| LM |  |
| no | 58(81.7%) |
| yes | 13(18.3%) |
| BM |  |
| no | 53(74.6%) |
| yes | 18(25.4%) |
| IM |  |
| no | 67(94.4%) |
| yes | 4(5.6%) |

ECOG-PS: Eastern Cooperative Oncology Group performance status;BM=bone metastasis; LM=liver metastasis； IM= Intracranial metastasis

Supplementary table 2 Uni-and multivariate analysis for PFS

| Parametres | Univariate analysis |  |  | Mutlivariate analysis | |
| --- | --- | --- | --- | --- | --- |
|  | OR (95% CI) | *p* |  | OR (95% CI) | *p* |
| HGF | 1.443(1.052-1.981) | 0.023 |  | |  |
| Sex | 1.023(0.562-1.863) | 0.940 |  |  |  |
| Age | 0.872(0.515-1.477) | 0.611 |  |  |  |
| Smoking history | 1.323(0.714-2.452) | 0.373 |  |  |  |
| ECOG-PS | 1.564(0.760-3.216) | 0.224 |  |  |  |
| T stage | 1.312(.522-3.297) | 0.564 |  |  |  |
| N stage | 1.991(1.179-3.363) | 0.010 |  |  |  |
| M stage | 2.806(1.662-4.740) | 0.000 |  | 2.679(1.560-4.600) | 0.000 |
| CEA | 1.007(1.002-1.011) | 0.002 |  | 1.006(1.001-1.013) | 0.010 |
| NSE | 1.005(1.001-1.009) | 0.015 |  |  |  |
| SCC | 0.834(0.591-1.176) | 0.300 |  |  |  |
| CYFRA21-1 | 1.063(1.001-1.129) | 0.046 |  |  |  |
| Pro-GRP | 1.000(1.000-1.000) | 0.256 |  |  |  |

ECOG-PS= Eastern Cooperative Oncology Group performance status; CEA=carcinoembryonic antigen ; NSE=neuron-specifific enolase; SCC:Squamous cell carcinoma antigen;CYFRA21-1=Soluble fragment of cytokeratin 19; Pro-GRP: pro-gastrin-releasing peptide
