# Supplementary material for: Plasma hepatocyte growth factor as a noninvasive biomarker in small cell lung cancer
Source: BMC Cancer. 2023 Oct 12;23:973. doi: 10.1186/s12885-023-10995-z (PMC10568809; doi:10.1186/s12885-023-10995-z)
Supplement: Supplementary file 2 — Supplementary Figure 1 [file 12885_2023_10995_MOESM2_ESM.docx]

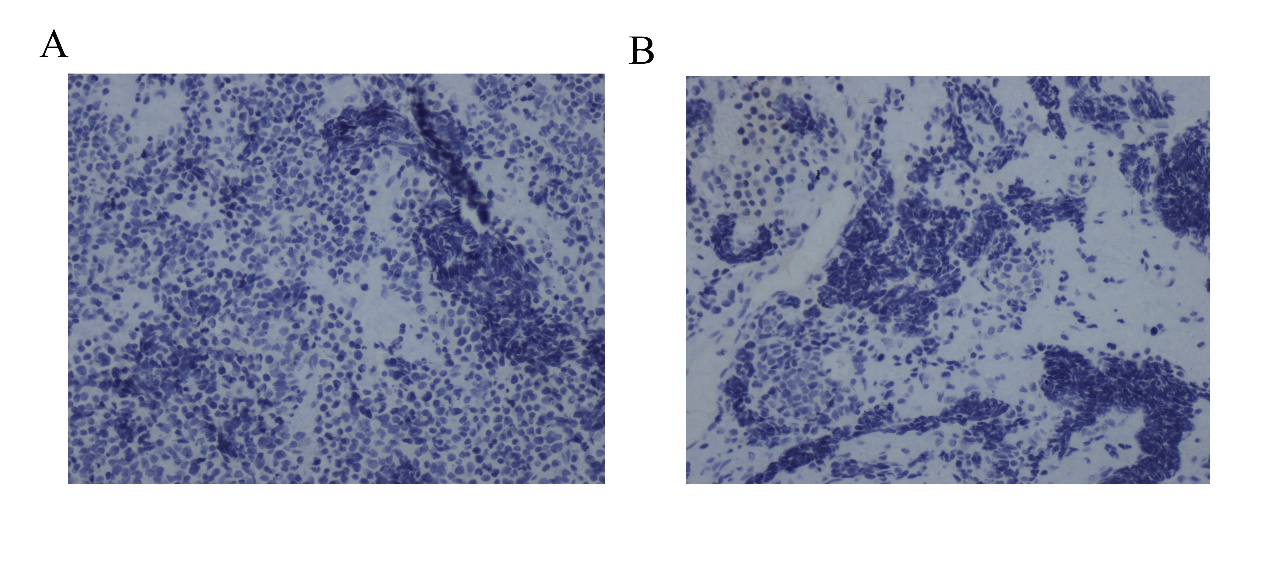


Supplementary Figure 1

SCLC are negative for HGF by immunohistochemistry, (original magnification ×20 in A-B)
